# Chemistry

**Published:** 1893-04

**Authors:** John A. Miller


### CHEMISTRY.

By JOHN A. MILLER, Ph. D.

### THE EXCRETION OF NITROGEN IN URINE.

Gumlich (*Zeit. Physiol. Chem.*, 17 ; *Jour. Chem. Soc.*, 62—1503). With different diets there were found :

1. A relatively large increase and diminution of urea with meat and vegetable food respectively.

2. A relative increase of ammonia with vegetable food. There was no change with animal food only.

3. A large relative diminution and increase of extractives with meat and vegetable diet respectively. A large number of morbid urines were also examined. In febrile disorders there was a relative lessening of urea, an increase of extractives and of ammonia. In diabetes, all cases showed a relatively high percentage of ammonia; the amount of extractives and urea, as in healthy persons, varied with the diet. In cases of liver cirrhosis, severe anemia, and heart failure, there was relative lessening of urea, and increase of ammonia and extractives. In cases of kidney diseases, there was a lessening of total nitrogen, and an absolute and relative increase of ammonia. In uremia the urea excreted varies but little, but the extractives almost disappear ; these substances appear to be retained in the tissues. The urea excreted in kidney disease is relatively great.

---

### PTOMAINES OF INFECTIOUS DISEASES.

Griffiths (*Compt. Rend.*, 114 ; *Jour. Chem. Soc.*, 62—1258.) (*Glanders.*)—The author has extracted from the urine of victims to glanders a ptomaine which forms white crystals of the composition $C_{15} H_{10} N_2 O_6$. The base is poisonous, and when injected under the skin of a rabbit, produces abscesses at the point of injection, peculiar nodular lesions in the lungs, etc., and finally death.

*Pneumonia.*— The ptomaine from the urine of pneumonia patients forms white, microscopic needles, soluble in water, forming an alkaline solution. It forms a hydrochloride, platinochloride, and aurochloride, and its solutions give a white precipitate with phosphotungstic acid, a yellowish white precipitate with phoshyphen phomolybdic acid, a yellow precipitate with picric acid, and a brownish precipitate with Nessler's solution. Neither of these ptomaines is found in normal urine.

---

### A NEW LEUCOMAINE.

Griffiths (*Compt. Rend.*, 115 ; *Jour. Chem. Soc.*, 62—1367,) reports that a considerable quantity of the urine of an epileptic was made alkaline with sodium carbonate, shaken with half its volume of ether, the filtered ethereal extract agitated with aqueous tartaric acid, and the alkaloid precipitated from the acid solution by excess of sodium carbonate. It was then taken up with a fresh quantity of ether, and on evaporating, the latter was left as white, oblique prisms. The *leucomaine*, $C_{12} H_{16} N_5 O_7$, thus obtained, is soluble in water, is feebly alkaline in reaction, and forms a crystalline by hydrochloride and amochloride. It is poisonous, producing trembling, intestinal, and urinary evacuations, dilatation of the pupils, convulsions, and death.

---

### INFLUENCE OF HOT BATHS ON THE EXCRETION OF NITROGEN AND URIC ACID FROM THE HUMAN SYSTEM.

Formanek (*Jour. Chem. Soc.*, Vol. LXII.—1503). A single hot air or vapor bath does not cause any perceptible change in the excretion of nitrogens from the system, but if baths be taken on two consecutive days, the excretion of nitrogen is found to increase on the second day, the increase continuing until the day following. The same thing holds also for the excretion of uric acid. A temporary raising of the temperature of the body is, apparently, therefore, of little effect in this respect, it being necessary to maintain the action for some time before change is noticed.

---

### ACTION OF OXALIC ACID AND ITS DERIVATIVES ON THE ANIMAL ECONOMY.

KROHL (*Chem. Centr.*, *I.*; *Jour. Chem. Soc.*, 62—1019.) Sodium oxalate, sodium malonate, ammonium oxalurate, and oxamide cause glycosuria, accompanied by an interruption of the oxidation processes, and carbon monoxide is formed, either in the free state or in combination. Since hydrogen cyanide (*prussic acid*) is oxidized to oxamide by *hydrogen peroxide*, experiments were made to see if it could be employed as an *antidote* in the case of prussic acid poisoning.

These experiments were successful, and *prussic acid*, in larger quantity than the fatal dose, was administered to dogs and cats, and its effects stayed by means of *hydrogen peroxide.* The experiment could be made daily for weeks together without permanently injuring the animal. Moreover, prussic acid could not be detected in the urine after application of hydrogen peroxide, whereas it is present in ordinary cases of prussic acid poisoning.

